# Comparative Performance of Three Magnesium Compounds on Thermal Degradation Behavior of Red Gum Wood

**DOI:** 10.3390/ma7020637

**Published:** 2014-01-24

**Authors:** Yiqiang Wu, Chunhua Yao, Yunchu Hu, Xiaodan Zhu, Yan Qing, Qinglin Wu

**Affiliations:** 1 College of Materials Science and Engineering, Central South University of Forestry and Technology, Changsha 410004, China; E-Mails: chunhuayao8@gmail.com (C.Y.); hucsfu@163.com (Y.H.); zhuxd0610@126.com (X.Z.); qingyan0429@163.com (Y.Q.); 2 School of Renewable Natural Resources, Louisiana State University AgCenter, Baton Rouge, LA 70803, USA

**Keywords:** magnesium compounds, fire retardant, wood, thermal degradation

## Abstract

The effect of basic magnesium carbonate (BMC), magnesium hydroxide (MH), and magnesium chloride hydrate (MCH) on thermal degradation of red gum wood was studied using cone calorimetry, Thermogravimetric-differential scanning calorimetry (TG-DSC) analysis, and X-ray diffraction (XRD) characterization. The results showed common fire retardation actions of the three compounds by releasing incombustible gas and/or water vapor to dilute combustible gas in the flaming zone, and by converting to MgO, which had a satisfactory protective wall effect on the wood. Individually, BMC absorbed heat from the wood at the pre-decomposition stage and, thus, slowed down wood pyrolysis process. It slightly increased the char yield by charring in both the charring stage and the char calcination stage. MH lost water at about 270°C, close to the temperature at which wood thermally degraded. MH rendered wood char quickly, and the compact char layer impeded further carbonization and burning of inner wood. MCH promoted charring with Mg^2+^ as a Lewis acid, and increased wood char yield. MCH also released Cl· free radical and HCl at 167°C, which easily coordinated with combustion reaction radical, and slowed down, even inhibited, the combustion chain reaction.

## 1. Introduction

Inorganic chemicals are attracting more and more attention among common fire-retardants due to their low cost and environmental-friendliness. Specifically, magnesium compounds are effective fire-retardants, and have been used for fire retardation treatment of polymers. For instance, magnesium hydroxide (MH: Mg(OH)_2_) has been used in polypropylene (PP), polyethylene (PE), and polyvinyl chloride (PVC) [1–4]. Magnesium chloride hydrates (MCH: MgCl_2_·*x*H_2_O), and basic magnesium carbonate (BMC: *x*MgCO_3_·*y*Mg(OH)_2_·*z*H_2_O) have also been reported to improve fire resistance of polymers [5–7].

Limited study has been reported on using these three magnesium compounds for fire retardation treatment of wood- and other cellulosic materials. Ondrej Grexa *et al*. [8] carried out a cone calorimetric study of MH-treated plywood, and showed that MH reduced specific extinction area (SEA—a smoke generation parameter) of wood from about 50 m^2^/kg to 40 m^2^/kg, and carbon monoxide yield from about 0.009 kg/kg to 0.007 kg/kg. MH suppressed smoke generation because its decomposition absorbed heat and diluted the flammable volatiles. Sain *et al*. [9] studied fire retarding effects of MH for sawdust filled PP composites and showed that the addition of MH reduced horizontal burning rate of the composites from about 33 mm/min to 15 mm/min, and raised their oxygen index (*i.e.*, minimum oxygen concentration required to support combustion) from about 20% to 34%. MH had a high endothermic value of 1450 J/g during its decomposition into magnesium oxide and water at 300–320°C. Shimada *et al*. [10] heated a mixture of cellulose and MgCl_2_ at 105°C for 24 h, and then conducted wide-angle X-ray diffraction (XRD) and gel-permeation chromatogram analysis on the products. A solid-state hydrolysis mechanism was proposed, which states that Mg^2+^ acted as a Lewis acid in solid-state MCH at a temperature of 105°C and was able to coordinate with the glycosidic oxygen from cellulose. Mg^2+^ then catalyzed the glycosidic bond cleavage, leading to increased yields of char [11]. Kawamoto *et al*. [12] carried out pyrolysis on MgCl_2_-treated filter paper and characterized the products. It was found that MgCl_2_ had catalytic action at 250°C on the polymerization of levoglucosan in cellulose and reduced the formation of volatile levoglucosan and flammable volatile yield. They also found that MgCl_2_ increased the primary char yield of cellulose heated at 400°C. Mostashari *et al*. [13] adopted thermo-gravimetric (TG) and vertical flame spread test to investigate the combustion and decomposition of cotton fabric coated with MCH. It was believed that the fire retardant action took place through gas dilution, chemical action, and free radical mechanisms. However, more experimental results are needed to confirm their fire retardant mechanisms for wood materials, especially for BMC.

The objective of this study was to reveal the fire retardancy effects of three magnesium chemicals (*i.e*., MH, MCH, and BMC) on wood with an emphasis on carbonization charring and chemical decomposition. A way of separating wood thermal degradation stages was outlined and thermo-kinetic calculations were carried out to obtain chemical reaction activation energy for each stage. The established fire retardancy effects of the chemicals can help better understand the interactions between wood and magnesium compounds to aid the selection of the chemicals as fire retardants.

## 2. Materials and Methods

### 2.1. Experiment Materials

Red gum (*Eucalyptus camaldulensis Dehnh.*) wood was used for the study. A clear air-dried red gum log was selected and processed to produce solid wood veneer and wood flour samples for subsequent tests. Wood flour with a 150-mesh particle size was made through grinding and screening. The flour was then mixed with each of the three compounds by a mass ratio of 85:15 and the mixture was ground in a grinding bowl to obtain homogeneous treated wood flour. The chemical compounds [*i.e*., MH: Mg(OH)_2_; MCH: MgCl_2_·6H_2_O; and BMC: 4MgCO_3_·Mg(OH)_2_·6H_2_O] were of analytical grade and used as received.

### 2.2. Cone Combustion Analysis

The cone calorimetric tests of the untreated and treated wood flour were performed on a Stanton Redcroft (Fire Testing Technology Limited, East Grinstead, UK) cone calorimeter following the ISO 5660-1 standard with pre-prepared wood flour samples. For each test, a sample of 14 g of untreated or treated wood flour was placed on the surface of a piece of aluminum foil inside a corundum crucible (100 mm length × 100 mm width), and the flour was firmly compressed to a uniform thickness. Subsequently, the crucible was mounted horizontally on the loader and exposed to the heat radiation of 50 kW/m^2^, which corresponded to a temperature of 780°C on the upper surface of the test sample.

### 2.3. Morphology Observation of Heat-Treatment Residues and XRD Characterization

For color and morphology observation, veneer samples with dimension of 10 (width) × 20 (length) × 0.7 (thickness) cm^3^ were separately impregnated with 0.1 mol/L magnesium compound suspensions for 12 h. The samples were dried and then heat-treated at each of the temperatures (167, 250, 300, 350, 430, 500, and 600°C) for 30 min to acquire the wood residues. Photographs of samples were taken with a digital camera for comparative analysis.

For powder XRD characterization, wood sample flour was calcined for 30 min in a muffle at a temperature of 500°C and then cooled in a glass desiccator. To avoid moisture absorption, all test samples were taken out of desiccators and loaded in the diffractormeter just several seconds before the tests. Powder XRD patterns were collected on a D/MAX 2550 VB/PC X-ray diffractormeter (Rigaku, Tokyo, Japan) using Cu Kα radiation of copper at ambient temperature with a working condition of 40 kV/250 mL. Diffraction peaks of crystalline phases were compared with those of standard compounds reported in JCPDS data file.

### 2.4. Combined TG-DSC Analysis

TG-DSC data of each of the untreated and treated wood flour, and pure chemical samples were simultaneously obtained using a STA449C (NETZSCH, Selb, Germany) coupled thermal analyzer. Each sample (~10 mg) was placed in a platinum crucible and heated at the rate of 15°C/min from ambient temperature to 650°C with dynamic carrier nitrogen gas flowed at a rate of 40 mL/min. Precured α-Al_2_O_3_ was used as a reference material. The mass fraction, *Y* (TG), the time derivative of the mass fraction, −d*Y/*d*t* (DTG) and the second time derivative of the mass fraction, −d^2^*Y*/d^2^*t* (DDTG) from the measured TG curves were calculated as a function of temperature and used for various analyses.

To calculate wood loss in the treated wood sample using the TGA data, mass loss of each corresponding chemical compound (measured from separated TGA runs) was subtracted from the mass loss of the treated wood samples using the following equation:

MLw(T1→T2)=(MAll1−MMc1×0.15)/0.85−(MAll2−MMc2×0.15)/0.85 (1)

where *MLw* (*T*1→*T*2), %, is the mass loss of the wood component in the treated wood sample from temperature 1 to 2, *MAll*1 and *MAll*2, %, are the mass losses of treated wood at temperatures 1 and 2, *MMc*1 and *MMc*2 are the mass losses of magnesium compound at temperatures 1 and 2. The values of 0.15 and 0.85 are the mass fractions of the magnesium compound and wood in the treated wood flour.

TG curves were further analyzed by a thermokinetic method to obtain the pyrolysis kinetic information. According to a previous study [14], Doyle approximate integration [15] was employed to model wood devolatiliztion, and Arrhenius equation was used to describe thermal pyrolysis rate. Thus, the linear thermokinetic equation of the wood pyrolysis is reduced to:

$$\text{ln}[-\text{ln}(1-\alpha )]=\text{ln}\left(\frac{AE}{\beta R}\right)-2.315-\frac{0.4567E}{RT}(n=1)$$ (2)

or

$$\ln\frac{{\left(1-\alpha \right)}^{1-n}-1}{n-1}=\ln\left(\frac{AE}{\beta R}\right)-2.315-\frac{0.4567E}{RT}(n\neq 1)$$ (3)

with the conversation rate of α expressed as:

$$\alpha =\frac{w_{0}-w}{w_{0}-w_{\infty }}\times 100\%$$ (4)

where *w*_0_, *w*, and *w*_∞_ are, respectively, sample weight at beginning, time, *t*, and end of each TGA run (mg), E is activation energy (kJ·mol^−1^); *R* is the universal gas constant (kJ·mol^−1^·K^−1^), A is the Arrhenius pre-exponential factor (s^−1^), and *T* is the pyrolysis temperature at time *t* (K). The values of 1, 1.5, 2, and 3 are acceptable reaction order *n* for the kinetics model of wood [14,16]. A linear regression analysis was done to obtain kinetic parameters using Equations (3) and (4).

## 3. Results and Discussions

### 3.1. Flame-Retardation and Smoke-Suppression Characteristics

**Figure 1.**
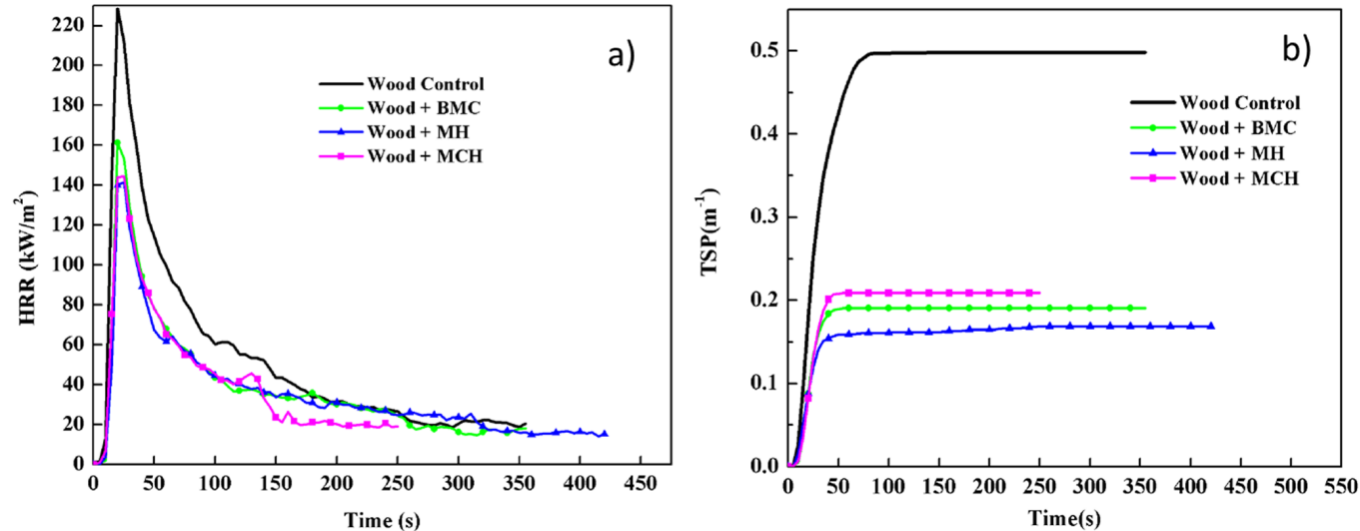
HRR (**a**) and TSP (**b**) as a function of time for untreated and magnesium treated red gum wood.

Figure 1 shows comparative plots of combustion parameters for untreated and treated red gum wood samples (a: heat release rate—HRR, and b: total smoke production—TSP). Table 1 lists summarized data. Test results showed that the three magnesium compounds were able to decrease the fire intensity and heat release of the wood during combustion, exhibiting satisfactory flame retardation effects. The HRR of the untreated wood was relatively high, which reached the peak value of 228.73 kW/m^3^ after being ignited. After treatment with magnesium compounds, the HRR values decreased by over 40%, and the treatment effects on HRR decreased in sequence of MH, MCH, and BMC. The total heat release (THR) values of treated wood were lowered by 20% to 40%. The magnesium compounds reduced TSP of wood by 58%–72%, total smoke rate (TSR) by 58%–66%, and average CO_2_ yield (CO_2_Y) by 7%–34%, while increased average CO yield (COY) by 20%–60%. Thus, the data showed evident smoke suppression effects of these three magnesium compounds. After fire-retardant treatment, COY for wood was increased while CO_2_Y was lowered, and the value of COY/CO_2_Y ratio increased (Table 1). Overall, the wood combustion process is not significantly changed. The slight reduction in HRR and the significant reduced smoke release indicated a slight gas phase mechanism, typically for barrier formation and fuel dilution.

### 3.2. Visual Observation of Thermally Treated Red Gum Wood

**Figure 2.**
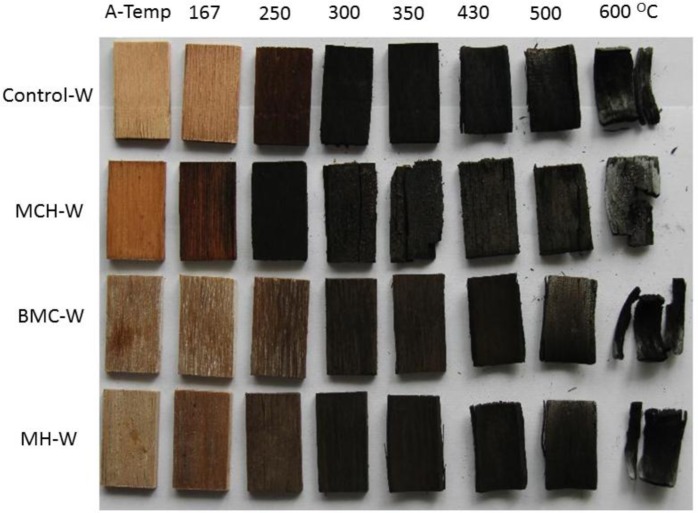
Photographs of untreated and fire-retardant treated red gum wood calcined at different temperatures.

As shown in Figure 2, with the proceeding of pyrolysis and charring, color and morphology of wood surface gradually changed. The wood control became dark reddish-brown at 250°C, and turned glossy black at 300°C. Considering the generally accepted wood thermal degradation onset temperature of about 260°C, the morphology of wood at 250°C indicated the start of wood degradation. Char structure of the control samples after 300°C was tight. The char started to warp when the temperature increased to 430°C and tar chips cleaved at 600°C.

MCH-treated wood became dark reddish brown at 167°C, and became glossy black at 250°C, indicating that pyrolysis and carbonization of the wood were brought forward. However, compared to the char structure of wood control, char structure of MCH-treated wood was coarser, and char started to cleave at 300°C. Gas generated from the combustion zone of wood at temperatures lower than 300°C, and the escape of gas made the char structure loose and easy to cleave. For BMC-treated wood, the gradual color variation from brown to black was slowed down compared to the control, and the treated wood did not completely turn black until the temperature reached 430°C. This decelerated pyrolysis process demonstrates a good fire-retardancy of BMC on wood. MH-treated wood became glossy black at 300°C, the same temperature as the control. However, the difference in color for the treated samples at ambient temperature and at 300°C was more obvious than that for the control. Thus, the pyrolysis process before 300°C was slowed down compared to the control, which indicate MH started to act before 300°C.

### 3.3. Chemical Identification of Heat-Treatment Residues

**Figure 3.**
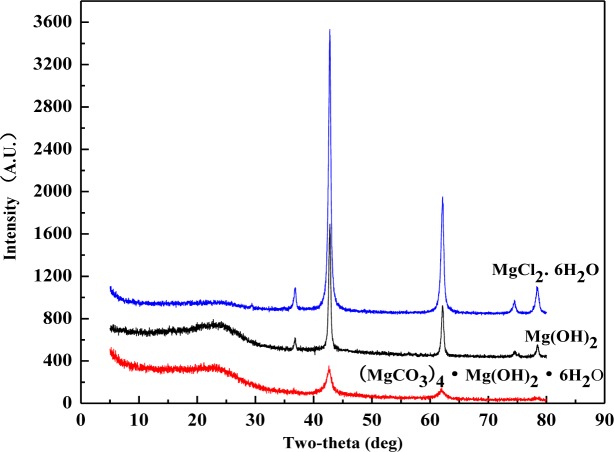
XRD patterns of treated red gm wood after being calcined at 500°C temperature.

XRD patterns were recorded for the wood samples calcined at 500°C after treated with magnesium compounds (Figure 3), and classic inorganic substances were specified by the diffractormeter database. XRD patterns of all these three sample flours demonstrated two main peaks at 2θ = 42.84° and 62.16°, which correspond to the peaks of pure MgO crystal substance. The database specified that the flour included no magnesium crystal rather than MgO. It indicates that elements C, O, H, and Cl in these magnesium compounds escaped in the form of gas before 500°C. Based on some previous research [9,17,18], gases produced by these magnesium compounds are nonflammable. These compounds displayed fire-retardancy by gas dilution mechanism. Furthermore, according to the dust or wall effect theory [19], the remained MgO in the consumed ashes can display flame-retardancy. The generation of MgO as an isolating protective layer was able to help the formation of coating over the surface of wood, leading to the extinction of flame. Therefore, all of these three magnesium compounds could display good flame retardancy by the mechanism of wall effect.

It should be pointed out that XRD experiment as shown was to demonstrate the existence of MgO in the wood residue from burning at lower temperatures. Theoretically speaking, all Mg compounds should be turned into MgO at about 500°C based on the current literature. Therefore, the XRD characterization was done with treated wood residue after calcination at 500°C. The cone calorimeter provided a heat radiation that corresponded to a temperature higher than 750°C. Under this temperature level, the MgO was also generated, and the XRD patterns from cone residue should be much the same as these shown in Figure 3.

### 3.4. Thermal Degradation Characteristics

**Figure 4.**
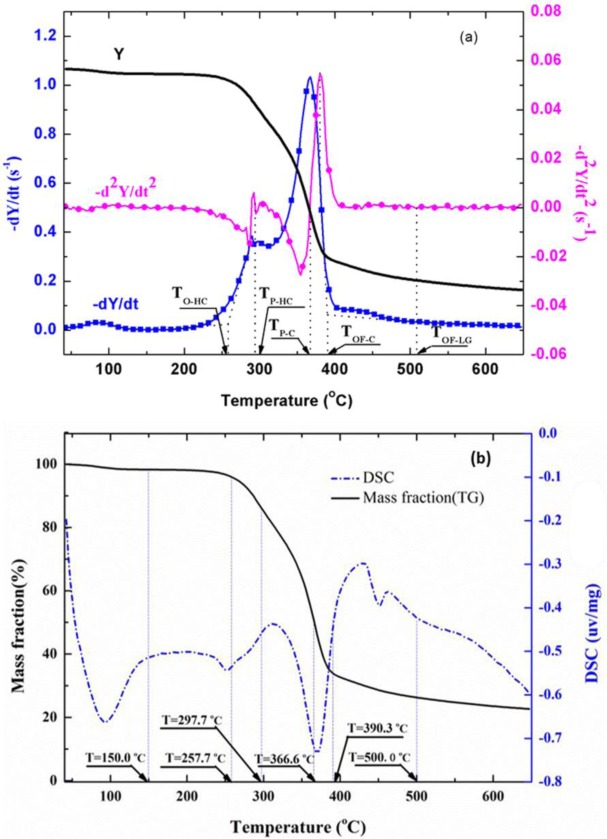
TGA and DSC data for untreated wood. (**a**) TG, DTG, and DDTG curves and definition of characteristic reaction temperatures; (**b**) combined TG and DSC curves and characteristic reaction temperature values.

The TG, DTG, and DDTG curves of untreated wood sample are shown in Figure 4. The DTG curves showed two main regions of decomposition in an agreement with previous findings [20,21]. It has been shown [22–24] that hemicellulose component in wood started to decompose at about 225°C and is almost completely degraded at around 325°C. Cellulose was more stable and mainly degrades at temperatures between 315°C and 400°C, and lignin degraded slowly over a long broad range from about 200°C to over 400°C. Therefore, the first peak region in DTG curve was mainly due to the decomposition of hemicellulose, and the second region was mainly from cellulose. Decomposition of lignin followed to the end of degradation process.

To accurately separate different phases along the observed wood thermal degradation curve, some characteristic temperatures were defined as shown in Figure 4a. T_O-HC_ indicates the onset (beginning) temperature of hemicellulose decomposition, defined by extrapolating the slope of the devolatilization rate in correspondence with the first local minimum in −d^2^*Y*/d*t*^2^ (down to the zero level of *Y* axis). *T*_P-HC_ is the peak temperature of the hemicellulose decomposition, identified by the point of −d^2^*Y*/d*t*^2^ = 0 in the first region. *T*_P-C_ is the peak temperature of the cellulose decomposition, identified by the point of −d^2^*Y*/d*t*^2^ = 0 in the second region. The beginning of the final and tailing region (also the end of the second region) dominated by lignin decomposition, is defined by the offset temperature, *T*_OF-C_, obtained by extrapolating the devolatilization rate corresponding to the local maximum in −d^2^*Y/*d*t*^2^ values. The offset temperature for lignin, *T*_OF-LG_, is obtained by extrapolating the devolatilization rate corresponding to the local maximum in −d^2^*Y*/d*t*^2^ values in the final region.

The obtained temperature values of *T*_O-HC_, *T*_P-HC_, *T*_P-C_, *T*_OF-C_, and *T*_OF-LG_ for untreated red gum wood are 150.0, 257.7, 297.7, 366.6, and 390.3°C, respectively (Figure 4b). The degradation process of the wood was separated into four stages from the established characteristic temperatures [14,16,23–25]. The first stage from the beginning to about 150°C was drying stage, in which moisture desorption, and softening and melting of wax inclusion occurred. The second stage was from about 150°C to around 250°C, for dehydration from cellulose unit and decomposition of the extractives (*i.e*., pre-degradation stage). The third stage was combustion and charring stage, which is characterized by fast volatilization of wood and flaming combustion (258 to 390°C). The last stage was char calcination stage with temperatures ranging from 390°C to 650°C. Oxidation of residual char after flaming combustion resulted in glowing ignition of the char in this phase.

**Figure 5.**
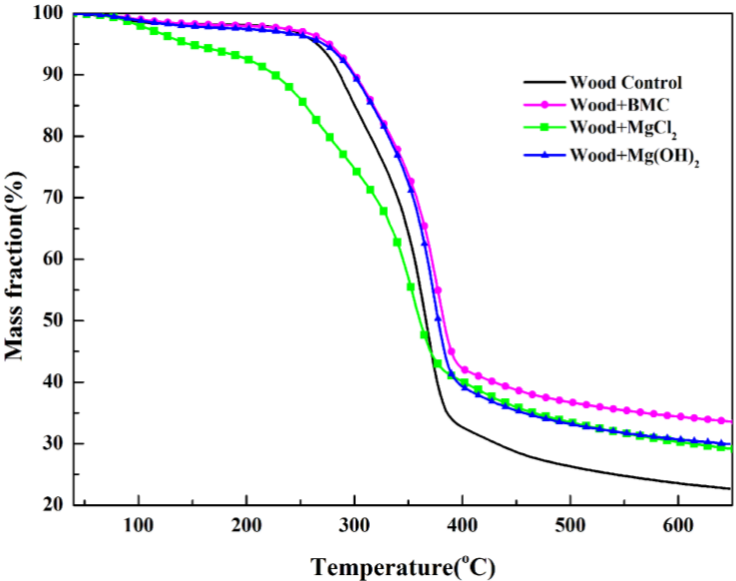
A comparison of TG curves of untreated and treated red gum wood.

To validate this separation method, measured heat flow DSC curve of untreated wood is shown in Figure 4b in comparison with the corresponding TG data. The heat flow curve showed an endothermic peak at around 100°C, resulting from the heat absorption of water evaporation. With the temperature increase, the heat flow value increased and an exothermic peak appeared. Subsequently, the curve fell down and an intensive endothermic peak appeared around 370°C. The intensive endothermic peak at 370°C indicated that decomposition of wood in this stage absorbed heat (energy). Then the second exothermic presented around 430°C. The first and second peaks represented heat releases in pyrolysis of hemicelluloses and lignin, respectively [26]. The charring process was highly exothermal whereas volatilization was endothermal. Hemicellulose and lignin pyrolysis generated much higher solid residues, and the exothermal peaks observed in hemicellulose and lignin pyrolysis could be attributed to the charring. The full decomposition of cellulose might be attributed to the quick devolatilization reactions, leading to very few solid residues left [26]. It is clear that the temperatures for endothermic and exothermic peaks were in a good agreement with temperature values indicated by TG analysis, validating the separation method used. The TG curves of untreated and treated red gum wood samples were plotted (Figure 5), and separated into different phases, with their characteristic temperatures listed in Table 2.

**Figure 6.**
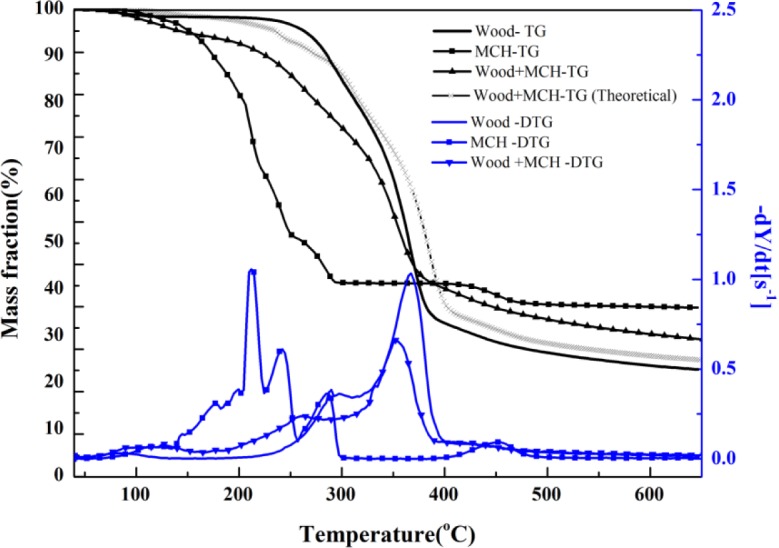
TG-DTG curves of untreated and MCH: MgCl_2_·6H_2_O-treated red gum wood.

MCH-treated wood started to degrade at around 130°C, and three obvious stages were presented on its DTG curve. The peak values were, respectively, around 133°C, 260°C, and 352°C, and a small shoulder around 340°C also presented. Based on the individual TG curves of MCH and wood, theoretical mass fraction data for MCH-treated wood was calculated and plotted according to the 15:85 mass ratios of MCH to wood (Figure 6). The experimental TG curve showed an obviously more mass loss than that from the theoretical curve from 150°C to about 380°C, indicating MCH started to act on wood at 150°C. Although the onset point of the second peak for MCH-wood was about 324°C, the region around 260°C, rather than 323°C, was regarded as the beginning of combustion and charring stage since the MgCl_2_·6H_2_O started to act at 260°C.

### 3.5. Fire-Retardant Action of Magnesium Compounds on Red Gum Wood

The calculated mass loss data of wood component in the treated red gum wood samples for each degradation phase using Equation (1) are listed in Table 3. The thermokinetic parameters calculated using Equations (2)–(4) with reaction order *n* = 1, 1.5, 2, or 3 for untreated wood control samples are summarized in Table 4 together with values of correlation coefficient. For the pre-decomposition and charring phase, reaction order of 1 led to better fitting result, and reaction order of 1.5 was better for the char calcination phase. Therefore, the kinetic parameters were calculated using reaction order of 1 for pre-decomposition and charring phase and 1.5 for char calcination phase for all treated samples and data are listed in Table 4. The calculated activation energy data values were in the general range of published data for fire retardant treated wood [14]. It is noted that the published activation energy data varied with analytical methods (e.g., reaction order), wood species, treatment chemicals, and temperature range selected.

#### 3.5.1. BMC-Treated Red Gum Wood

At the pre-decomposition stage (150.0–260.5°C), both mass loss of the wood component and activation energy of the treated red gum wood sample were reduced compared to these of the wood control. BMC was reported to have first degradation endothermic peak between 130 and 350°C, and it thermally decomposes, losing H_2_O and generating MgCO_3_, MgO, or dehydrated basic magnesium carbonate at a temperature of lower than 250°C [27–30]. Therefore, the decreased mass loss for wood component in the treated sample at the pre-decomposition stage was a result of BMC, itself, absorbing heat, reducing the amount of heat from the wood component. The reduced activation energy in this region was due to the decomposition of BMC itself rather than the wood component. Compared with wood control, TG curve of the wood component showed no obvious difference during this pre-decomposition phase. The wood residue photographs of the treated wood (Figure 2) also indicated no obvious variation at temperatures lower than 250°C, further illustrating that BMC treatment at this stage made no difference in the pre-decomposition behavior of wood.

Compared with the wood control, wood component of the treated samples had a slightly reduced mass loss at the combustion and charring phases, and an increased mass loss at the char calcination stage. Meanwhile, the treated sample had higher activation energy at combustion and charring stage and a decreased energy value at the char calcination stage. The char yield rate for wood component was slightly higher than that of the wood control. The wood residue photograph for the treated wood showed no obvious difference from the wood control. All of the evidence indicated that BMC had the ability of promoting charring. However, charring was not completely over at the combustion and charring stage. At this stage, the elevated activation energy indicated that the absolute change rate of reaction rate constant *k* to temperature (d*k*/d*T*) was reduced down, and this helped push back the charring process. Therefore, in the combustion and charring phase, the temperature was not increased to a level of high enough to accumulate the required energy for the charring of all wood, and some wood was left for charring during the next temperature zone. Combustion residue photograph (Figure 2) showed that the treated wood sample started to turn glossy black at 430°C while the wood control became glossy black at 300°C. It proved that the treated wood had a much slower charring process. The decreased activation energy at char calcinations process for the BMC treated wood indicates that char calcination reaction was easier to start compared to the control. This lowered activation energy was possibly caused by the further charring of wood, which was not previously fully carbonized.

#### 3.5.2. MH-Treated Red Gum Wood

MH started to lose water molecules and convert to MgO from about 270°C [31]. This temperature level is close to the reported temperature at which whole wood tends to thermally degrade. Therefore, MH exerted fire-retardant effects on wood by diluting the combustible gas. At the pre-degradation stage, the wood component of treated sample had an increased mass loss and the treated sample had a significantly reduced activation energy value compared to the wood control. This illustrates that MH might promote wood pre-pyrolysis at this stage as more wood tended to pre-decompose compared to the control.

Data in Table 3 show that mass loss increased at the combustion and charring stages and reduced at char calcination stage, while activation energy increased at both stages. The color difference of wood residues (Figure 2) between 250 and 300°C for MH-treated wood was more obvious than that for the wood control. It demonstrates that MH was able to boost wood charring in a short time, and brought higher mass loss at this point. The char layer of the calcinated MH treated wood was compact, and was able to impede the release of heat and gas and hinder the further calcination of char layer at the next stage. Thus, the mass loss at char calcination stage was reduced and the activation energy at this stage was raised. The increased activation energy at charring stage indicated that the absolute change rate of reaction rate constant to temperature (d*k*/d*T*) was reduced and the charring was unable to proceed as the temperature raising. When the temperature was raised to a high enough level, charring started and proceeded with a high reaction rate, and well-defined glossy black char was generated (Figure 2).

#### 3.5.3. MCH-Treated Red Gum Wood

Multi-step thermal decomposition occurred for MCH at the temperature levels of about 130, 160, 200, 250, 290, and 430°C. MCH gradually lost the hydration water from about 130°C, and started to convert to MgOHCl and released HCl at the temperature of 160°C. In a [H_2_–O_2_] system like wood combustion, the propagation of combustion was due to the reaction of ·H + O_2_ → ·OH + O· and O· + H_2_ → ·OH + ·H, and heat was released by the reaction of ·OH + CO → CO_2_ + ·H. The HCl generated coordinated with ·H and O·, and produced Cl· as follows: ·H + HCl → H_2_ + Cl·, ·OH + HCl → H_2_O + Cl·. Meanwhile, MgOHCl also released Cl·, which was less active radical than H and O. Therefore, both pathways slowed down or even terminated the combustion chain reaction.

The pathway of MCH losing its hydration water molecules and chloride radicals in the combustion propagation zone is verified by the generation of MgO according to the XRD results (Figure 3). Since MgO was the only detected magnesium compound, it can be concluded that the decomposition of MCH completed at the temperature of 500°C. In terms of mass loss and activation energy, wood component in treated wood sample had a lower mass loss at combustion and charring stage and higher loss at the char calcination stage compared to the control. The treated wood and the control had much the same mass loss at the pre-decomposition stage.

The char layer of MCH-treated wood generated at a relative early time and temperature less than 250°C, and the TG curves clearly showed that charring stage for MCH-treated wood started from about 223°C. Therefore, it can be inferred that charring was brought forward and the compact char layer was generated on the wood substrate surface at an early time, preventing the inner part of wood from burning and charring. In this way, the mass loss at charring stage was reduced. Therefore, it suggests that MCH catalyzed charring at the early time of the charring stage. However, with the proceeding of combustion, when increasingly more gas evolved from MCH treated wood and gradually went through the tight charring layer, the structure of char layer became coarser even porous. Charring continued when the char structure became loose then. Variation in the mass loss can be justified if the charring of the treated wood ever suspended at the later zone of charring stage, and restarted at the char calcination stage.

The above assumption is found to be reasonable considering decomposition of MCH and its interaction with cellulose. According to the previous research [10,14], solid-state MCH as a fire-retardant for cellulose can coordinate with the glycosidic oxygen, catalyze the glycosidic bond cleavage around 105°C and lead to an increased yield of char. Therefore, it is possible that MCH started to catalyze charring at the beginning of charring stage, and compact char layer further slowed pyrolysis process. In addition, based on another study [18], MCH started to releases crystal water at 69°C, and generated MgOHCl and HCl at the temperature range of 167°C to 415°C. The release of large quantity of gas helped loosen the structure of char layer, and then lead to the charring of wood at the char calcination stage.

## 4. Conclusions

Comparative performance of three magnesium compounds on the thermal degradation of red gum wood was analyzed in terms of mass loss, char yield, activation energy, and wood residue chemical components and morphology. All three compounds were able to release incombustible gas and/or moisture to dilute the combustible gas in the flaming zone. At the same time, the chemicals were converted to MgO before 500°C, and the MgO had satisfactory protective wall effects on wood.

Individually, BMC in the treated wood samples absorbed heat during the pre-decomposition stage (temperature less than 250°C), slowing down wood combustion and charring process. The release of water vapor and CO_2_ from BMC at the combustion and charring stage led to formation of loose char layer, and inner wood was subject to further charring at the char calcinations stage. MH started to lose absorbed water and to convert to MgO at about 270°C, which slowed down the pre-decomposition rate at temperatures lower than 300°C. MH boosted wood charring at the beginning of charring stage, and the formed compact char layer impeded the further carbonization and burning of inner wood. For MCH-treated wood, Mg^2+^ as a Lewis acid in solid-state MCH coordinated with the glycosidic oxygen, and catalyzed the glycosidic bond cleavage, thus brought forward onset of charring stage to around 222°C and desirable char layer generated at the charring stage. The char layer protected inner wood from pyrolysis for a certain period of time. However, release of hydrochloride and water moisture from inner part of wood with further increase of temperature led to formation of loose and porous char layer and further charring of inner wood at the char calcination stage. In addition, Cl· free radical and HCl were generated at 167°C, and they easily coordinated with combustion reaction radicals of ·H and O·, therefore slowed down even terminated the combustion chain reaction.

## Figures and Tables

**Table 1.** Values of combustion parameters for untreated and treated red gum wood samples.

Sample *	THR (MJ/m^2^)	TSR (m^2^/m^2^)	Average COY (kg/kg)	Average CO_2_Y (kg/kg)
W	18.71	56.16	0.05	1.38
WBMC	13.96	21.52	0.06	1.28
WMH	14.85	19.04	0.08	1.25
WMCH	10.99	23.55	0.06	0.91

* W—Wood only; WBMC—Wood + Basic Magnesium Carbonate; WMH—Wood + Magnesium Hydrate, WMCH—Wood + Magnesium Chloride Hydrate.

**Table 2.** Degradation characteristics temperatures of untreated and treated wood.

Sample*	De-hydration (°C)	Pre-decomposition (°C)	Combustion and charring (°C) **						Char calcination (°C)
			*T*_O-I_	*T*_P-I_	*T*_O-HC_	*T*_P-HC_	*T*_P-C_	*T*_OF-C_	
W	40.0–150.0	150.0–257.7	–	–	257.7	297.7	366.6	390.3	390.8–650.0
WBMC	40.0–150.0	150.0–260.5	–	–	260.5	318.5	377.6	390.8	390.8–650.0
WMH	40.0–150.0	150.0–265.2	–	–	265.2	329.7	374.7	393.3	393.3–650.0
WMCH	40.0–150.0	150.0–222.5	222.5	262.1	323.6	333.0	352.6	381.4	381.4–650.0

* W—Wood only; WBMC—Wood + Basic Magnesium Carbonate; WMH—Wood+ Magnesium Hydrate, WMCH—Wood + Magnesium Chloride Hydrate

** O—onset, P—peak, I—initial, HC—hemicellulose, OF—offset, C—cellulose, and *T*—temperature.

**Table 3.** Mass losses and char yield of wood component in the treated red gum wood sample.

Sample*	Mass loss at different phases				400°C Char yield (%)
	Dehydration (%)	Pre-degradation (%)	Combustion and charring (%)	Char calcination (%)	
W	1.72	2.18	62.10	10.90	32.56
WBMC	1.96	1.80	60.87	11.90	32.75
WMH	2.36	2.35	63.08	8.64	31.58
WMCH	5.26	0.00	52.42	14.43	39.93

* W—Wood only; WBMC—Wood + Basic Magnesium Carbonate; WMH—Wood + Magnesium Hydrate, WMCH—Wood + Magnesium Chloride Hydrate.

**Table 4.** Thermokinetic parameters for pyrolysis of fire-retardant treated red gum wood.

Sample *	Pre-degradation			Combustion and charring			Char calcination		
	*E* (kJ·mol^−1^)	*A* (s^−1^)	*r*^2^	*E* (kJ.mol^−1^)	*A* (s^−1^)	*r*^2^	*E* (kJ·mol^−1^)	*A* (s^−1^)	*r*^2^
W	221.6	1.1 × 10^9^	0.98	258.6	6.0 × 10^8^	0.95	186.7	9.2 × 10^4^	0.95
WBMC	144.4	6.0 × 10^5^	0.95	268.0	1.0 × 10^9^	0.95	157.0	1.6 × 10^4^	0.96
WMH	143.2	5.3 × 10^5^	0.96	282.8	3.3 × 10^9^	0.96	189.8	1.1 × 10^5^	0.94
WMCH	172.5	1.4 × 10^7^	0.98	111.7	1.2 × 10^4^	0.72	179.1	5.2 × 10^4^	0.95

* W—Wood only; WBMC—Wood + Basic Magnesium Carbonate (4MgCO_3_·Mg(OH)_2_·6H_2_O); WMH—Wood + Magnesium Hydrate, WMCH—Wood + Magnesium Chloride Hydrate.
